# A Comparison of Clinical Outcomes Between the Winograd and Modified Winograd Methods for Ingrown Toenails: A Retrospective Study of the Importance of Suturing Techniques

**DOI:** 10.7759/cureus.25941

**Published:** 2022-06-14

**Authors:** İbrahim Altun, Gökhan Peker

**Affiliations:** 1 Orthopaedics and Traumatology, Kayseri City Hospital, Kayseri, TUR; 2 Orthopaedics and Traumatology, İstinye University, Trabzon Yıldızlı Medicalpark Hospital, Trabzon, TUR

**Keywords:** recurrent ingrown toenails, knot tecnique, surgical treatment, modified winograd method, ingrown nails

## Abstract

Aim: This study aimed to compare the effects of the Winograd and modified Winograd methods for nail bed suturing on clinical outcomes in patients with nail ingrown.

Methods: In total, 45 patients who underwent surgery for ingrown toenails between December 2019 and December 2020 were randomised retrospectively. In this study, different methods applied by the authors were studied, and the methods applied by each author were divided into two separate groups. All patients had partial germinal matrix and nail bed excisions. Thirty (53.6%) of the nails were dressed in gauze, leaving the excised area of ​​the nail unsutured (group 1). Then, the remaining 26 nails (group 2) were sutured with the mattress suturing technique to ensure that the skin was under the nail. Clinical outcomes, visual analogue scale (VAS) scores, and verbal satisfaction status were statistically evaluated.

Results: In our study, 56 nails of 45 patients were evaluated retrospectively. The patients were followed up for an average of 13 (10­19) months. The mean age was 27.13 (15-­48) years. In total, 31 (68.9%) of the patients were men, and 14 (31.1%) were women. The incidence of postoperative bleeding and granulation tissue development decreased in the sutured group. In group 1, the development of hypertrophic granulation tissue after surgery had a significantly negative impact on VAS score and recovery time. In the sutured group, patients returned to work or performed activities of daily living for a shorter period. Approximately 95% of patients were satisfied or extremely satisfied.

Conclusion: Partial matrix excision using the appropriate suturing technique is associated with a fast recovery, low recurrence rate, high patient satisfaction, and earlier return to work activities of daily life among patients treated for ingrown toenails.

## Introduction

An ingrown toenail is a common and painful condition that leads to job loss, and it affects adolescents and young adults [1,2]. This pathology is caused by various factors such as faulty nail cutting, abnormally shaped nails and nail folds, excessive sweating, wearing tight shoes, poor foot hygiene, excessive body weight, hereditary factors, trauma, fungal infection, and anatomical predisposing factors, or a combination of these [1-3].

Patients with ingrown toenails are often evaluated with the Heifetz classification system. The condition is classified as follows: stage 1, the nail fold on the side turns into the nail bed; stage 2, there is discharge accompanied by acute and active infection; and stage 3, there is granulation tissue and hypertrophy in the nail fold with chronic infection [4]. In terms of therapy, patients with type 1 and partially type 2 ingrown toenails who are compliant can receive conservative treatment. However, surgical treatment is preferred for Heifetz type 2 and 3 ingrown toenails, particularly in patients whose pain does not resolve, thereby causing limitations in activities of daily living; those who are not successfully managed with conservative treatment; and those who are not applying conservative treatment [2-7].

Surgical methods used for ingrown toenails commonly include excision of the nail bed and the germinal matrix of the nail in this region [7]. The Winograd method was first described in 1929 and was used in our study. It is an effective treatment option for ingrown toenails because the nail bed and germinal matrix are resected [7]. Although it is effective, recurrence and cosmetic problems may develop. To obtain more effective outcomes using this method and to reduce the incidence of recurrence, different suturing techniques or other methods (such as cryotherapy, chemical or electrocauterization, and CO_2_ laser) are applied to the nail bed after excision [7-11].

This study aimed to evaluate the effect of suturing techniques applied to the nail margin on clinical outcomes among surgically treated patients with stage 2 and 3 ingrown toenails according to the Heifetz classification.

## Materials and methods

This study was approved by the local ethics committee of the institution. In total, 45 patients (56 nails) who presented to our outpatient clinic due to complaints of discharge, deformity, and pain in the big toenail between December 2019 and December 2020 were randomised and retrospectively evaluated. Thirty-two patients with recurrent ingrown toenails, diabetes mellitus, acutely infected nails, and structural nail disorders were excluded from the study.

Toes were divided into two groups: those with sutured and non-sutured nails to evaluate the effect of the suturing technique on clinical outcomes after excision, as described by Winograd. In this study, different methods applied by the authors were studied, and the methods applied by each author were divided into two separate groups. Patients with a pressure dressing but without a primary suture were followed-up and were included in group 1. Meanwhile, those whose nail edge on the skin was primarily managed with mattress suturing were classified under group 2. Clinical outcomes and visual analogue scale (VAS) scores were evaluated for two to three weeks to detect pain when they returned to work. Patient satisfaction and dissatisfaction were categorised verbally with a score of 1 indicating low and 5 indicating high satisfaction three months after surgery. Then, they were evaluated statistically.

Surgical technique

**Figure 1:**
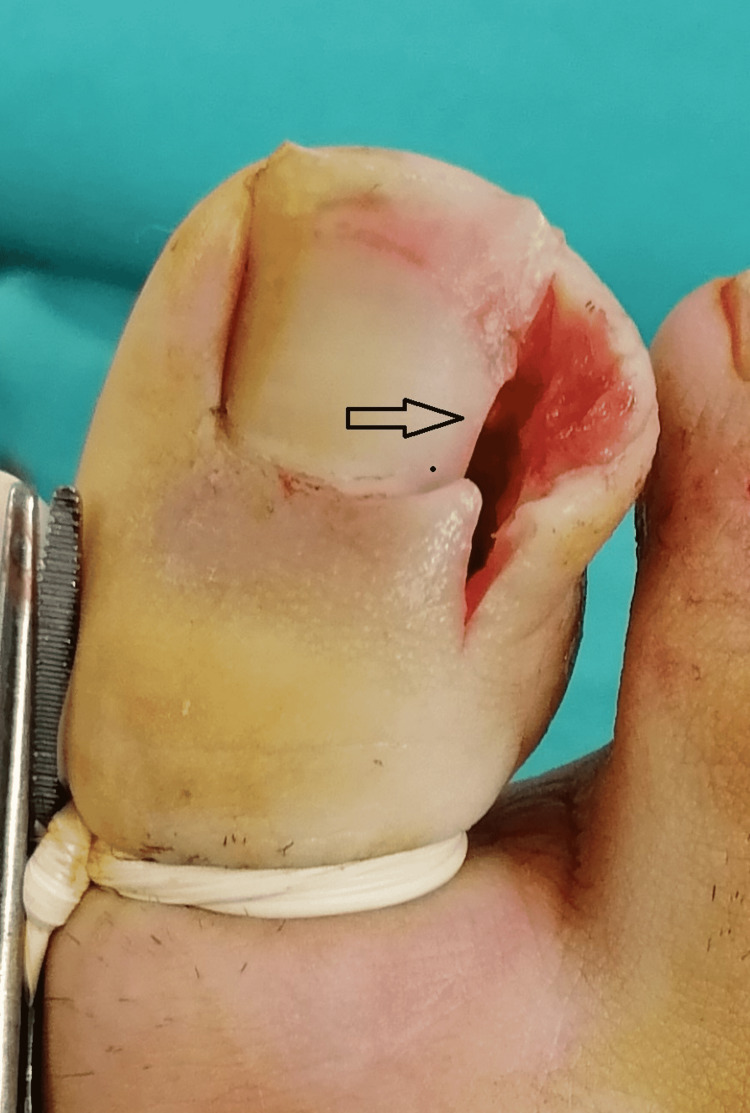
View of hypertrophic tissues and nails that were excised

In both surgical techniques, adrenaline-free lidocaine was used, and digital block anaesthesia and a finger tourniquet were applied. To prevent forgetting the tourniquet at the end of the procedure, it was fixed with a clamp. First, the hypertrophic tissues covering the nail were excised to the lateral nail fold. Then, the nail was cut vertically up to the phalanx, extending 5-10 mm proximal from the nail skin border to the nail lateralis. Starting from the proximal part, the whole nail edge was first resected. Then, the whole nail bed and the nail matrix were excised by stripping subperiosteally with the reverse side of the no. 11 scalpel over the phalanx (Figure 1).

**Figure 2:**
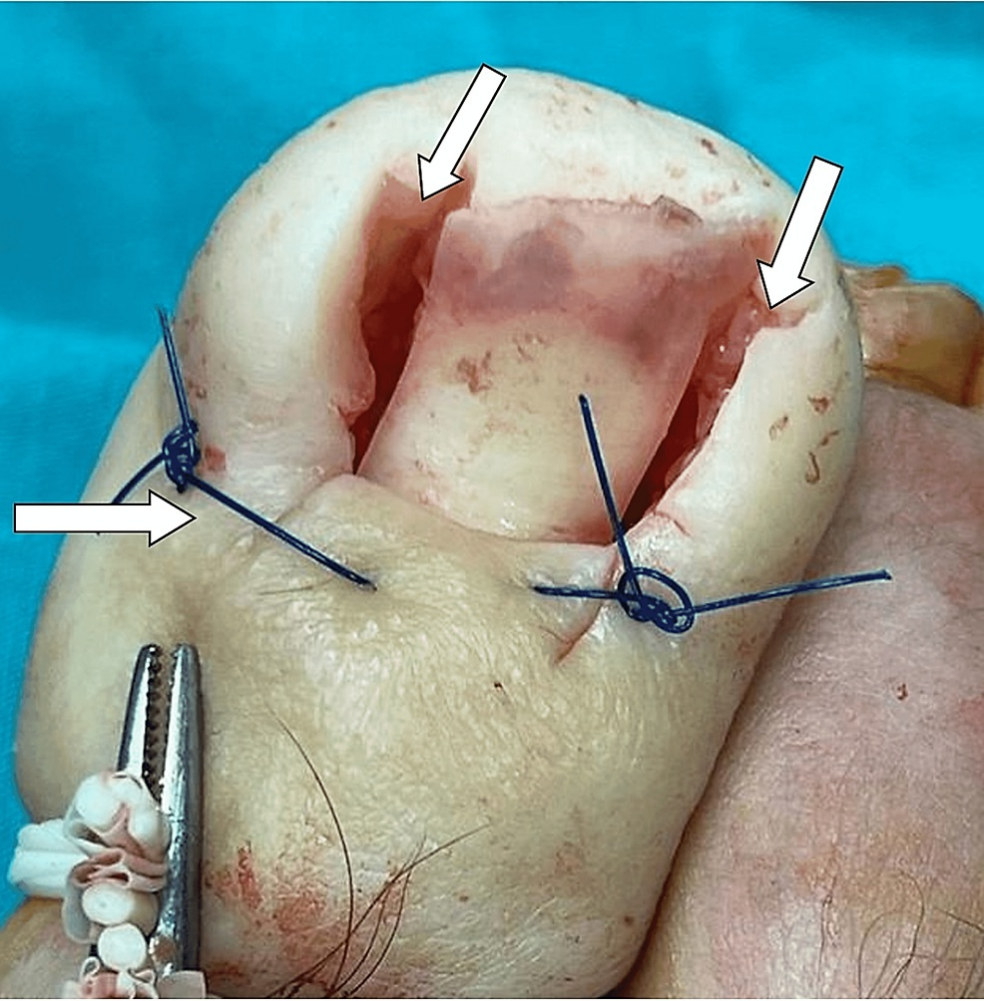
The appearance at the end of the surgery in the patients in group 1

If there was any residue, it was cleaned with a curette. The wound was washed with saline. No. 3 proline was used in the sutured areas in all patients. In patients in group 1, whose excised area was left open, only the skin in the nail matrix was closed, and the area around the nail was left empty, dressed, and closed (Figure 2).

**Figure 3:**
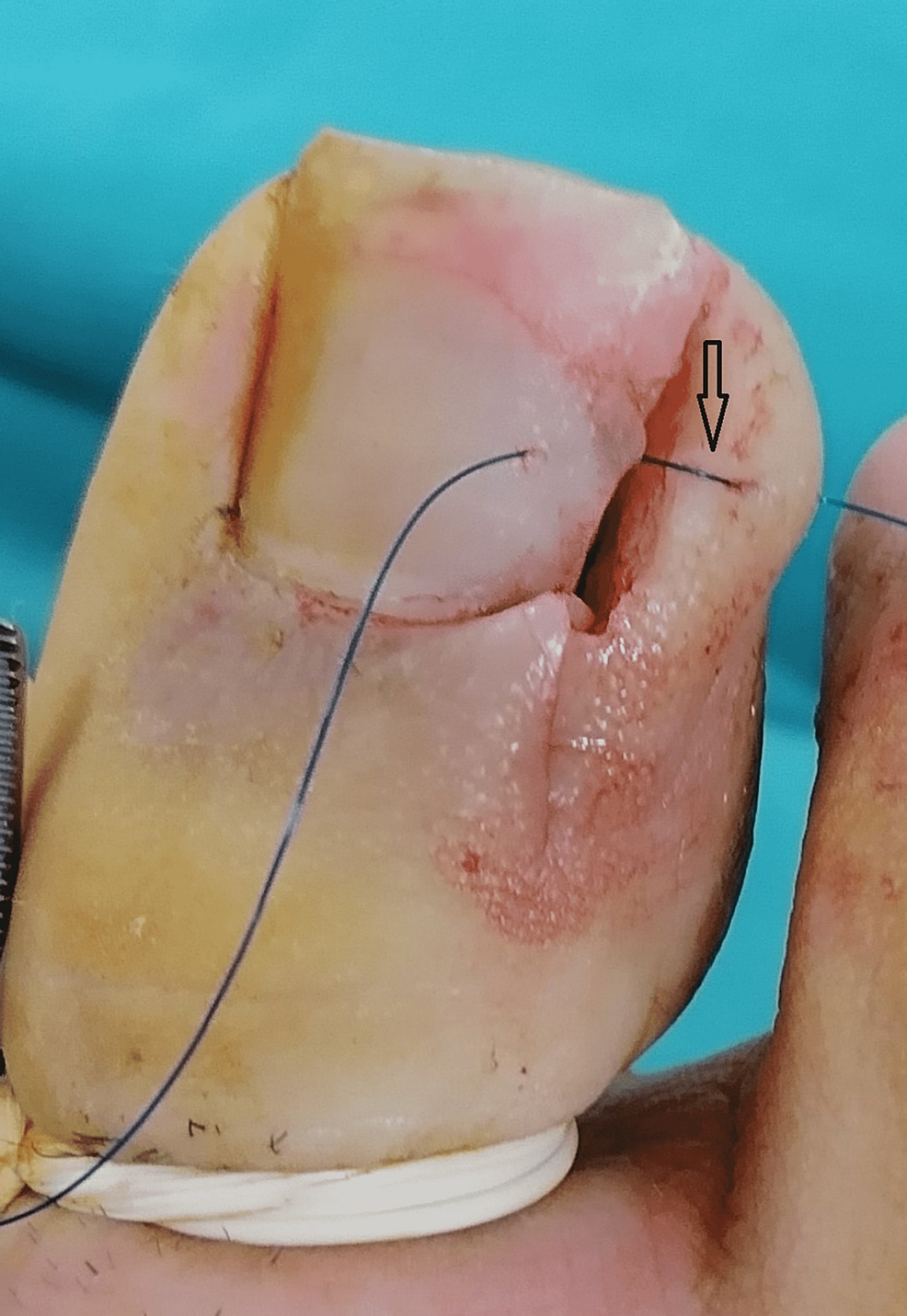
View during surgery, how the skin suture is placed in group 2

**Figure 4:**
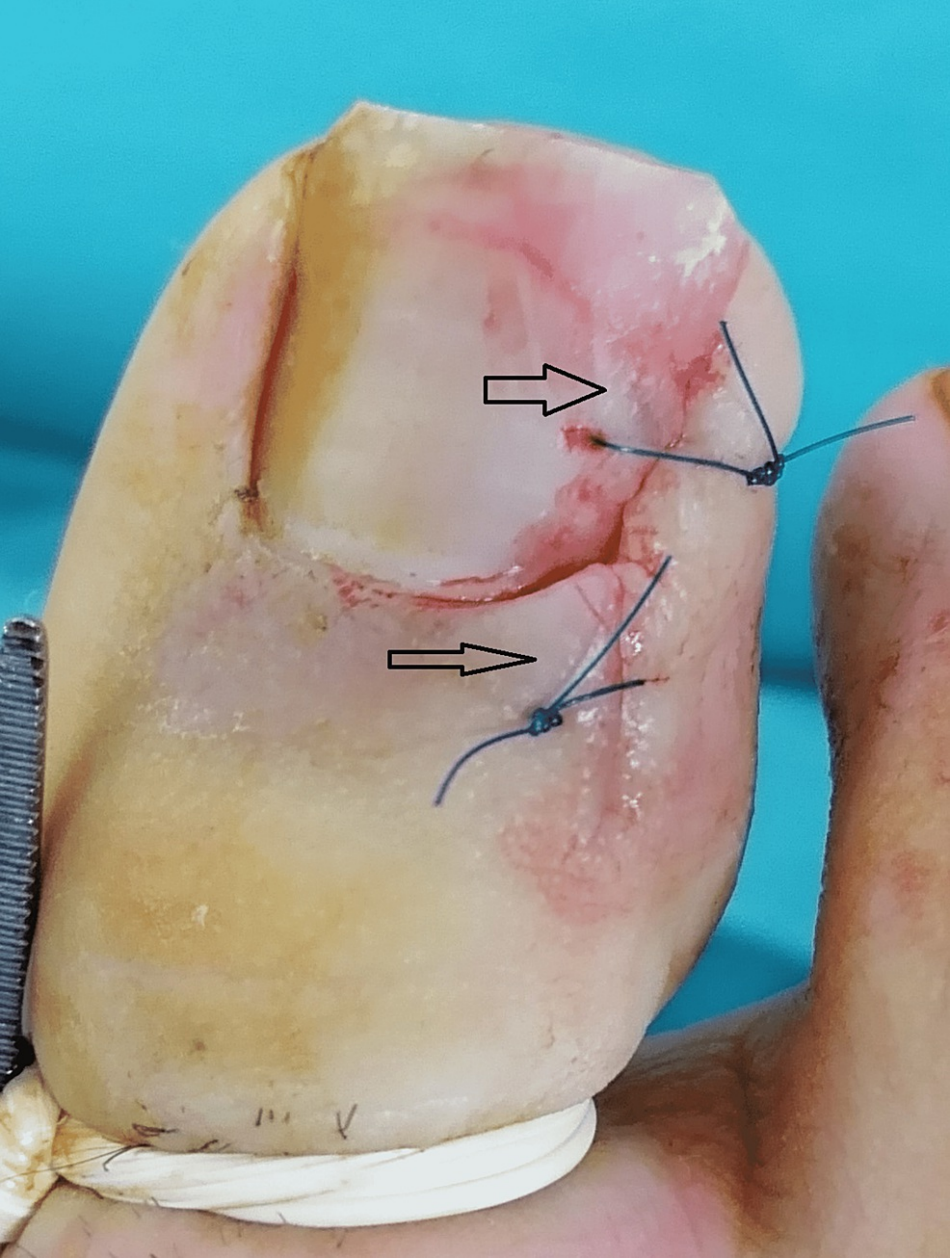
Postoperative view of the surgical field in group 2

In patients in group 2, a single suture was made with the matrix technique to ensure that the skin edge remained under the nail (Figure 3). After that, the excised area was closed and the procedure was discontinued (Figure 4).

Oral nonsteroidal anti-inflammatory drugs and 500 mg sodium fusidate twice a day for three days were used after the operation. The dressing was opened on the first post-operative day, and it was evaluated every two days until the stitches were removed (an average of 11 to 14 days). Recurrence follow-up was done every two months for one year.

Statistical analysis

After transferring the data to a computer, they were evaluated with the Statistical Package for the Social Sciences software (IBM Corp. Released 2013, IBM SPSS Statistic for Windows, Version 22.0, Armonk, NY: IBM Corp.). The normality of data was evaluated using the Shapiro-Wilk test and skewness and kurtosis values. To perform between-group differences, the Pearson chi-square test was used for categorical data and the independent sample t-test for continuous data. A p-value of <0.05 was considered statistically significant.

## Results

The patients were divided into two groups. In group 1, 53.6% (n = 30) of nails were left unsutured. In group 2, 46.4% (n = 26) of nails were sutured. The average age of the patients was 27.13 (15-48) years. In total, 28 toes in 50% of the patients had stage 2 ingrown toenails, and the remaining nails were stage 3. In total, 31 (68.9%) patients were men and 14 (31.1%) were women. Patients were followed up for at least 10 (average: 13) months. Sutures were removed in an average of 12 (11-14) days (Table 1).

**Table 1:** Demographic data of the patients and distribution according to the Heifetz classification *Independent t-test, Chi-square test, p≤0.05

	Group 1; n=30	Group 2; n=26	p-value
Age	28.9± 8.1	25.4± 5.6	0.094^*^
Sex			
Female	7	7	1.000^*^
Male	15	16	
Side			
Right	12	7	
Left	8	7	0.789^*^
Bilateral	5	6	
Active infection before surgery			
Yes	17	16	0.789^*^
No	13	10	
Heifetz			
Type 2	11	17	0.060^*^
Type 3	19	9	

Some patients developed hypertrophy of the granulation tissue within the first three weeks after surgery. In group 1, 80% (n = 24) of the nails developed mild (n=15), moderate (n=7), and severe (n=2) granulation tissue. Meanwhile, in group 2, 20% (n=5) of patients had mild granulation tissue. Patients with granulation tissue recovered with systemic antibiotic therapy without additional treatments. Only 3.6% (n = 2) of patients in group 1 experienced recurrence, which occurred in the fourth month after surgery. The toes with recurrence in group 1 were re-operated with the same method and healed uneventfully. None of the patients developed osteomyelitis or any other serious complications.

In our study, according to the chi-square test, the incidence of bleeding and granulation tissue in the post-operative period was significantly lower in group 2 (p<0.001). The prolongation of recovery time had a significantly negative effect on VAS values ​​(p<0.001). Based on the t-tests performed, the average times for return to work were 15.1 days in group 1 and 12.9 days in group 2, and the results significantly differed. However, there was no significant difference in VAS scores in times for return to work. In group 1, the development of granulation tissue during the post-operative period had a negative effect on recovery time (p<0.001; Table 2).

**Table 2:** Comparison of post-operative functional outcomes between groups *Chi-square test, independent t-test, p≤0.05

	Group 1	Group 2	p-value
Post-surgical bleeding			
Yes	30	2	<0.001^*^
No	0	24	
Granulation			
Yes	24	5	0.015^*^
No	6	21	
Recurrence			
Yes	2	0	0.211^*^
No	28	26	
Return to work (days)	15.1 ± 2.1	12.9 ± 1.3	<0.001^*^
VAS score (/10)	7.36 ± 0.9	7.61± 1.3	0.478^*^

## Discussion

An ingrown toenail is a painful disease that generally affects young adults and limits activities of daily living [1,2]. Surgical intervention is often required for ingrown toenails that are resistant to conservative treatment or that do not improve [2]. In surgical intervention, the Winograd or modified Winograd method is commonly used [7,8]. Partial or wedge matrix excision is preferred, and it has been performed by applying different suturing techniques or without suturing [9-15].

The rates of recurrence after surgery vary [11,13,15]. Çamurcu et al. showed a recurrence rate of 7.9% in 189 on-sutured nails that were managed using the original Winograd method [10]. In the study of Köse et al., the recurrence rate after wedge matrix excision in 75 nails and closure of the skin with the classical method was 13.2%. In the same study, cosmetic problems occur due to narrowing of the nail bed with wedge matrix resection [12]. However, with partial matrixectomy, which was applied in our study, the incidence of cosmetic problems was low, and patient satisfaction was as high as 95%.

Some researchers claimed that only nail matrix excision is associated with a high recurrence, and it causes cosmetic problems due to the proximal incision [12,16,17]. Therefore, methods such as the use of phenol, CO_2_ laser, chemical cauterisation, cryotherapy, and electrocauterization have been used along with matrix resection [16-19]. In the study of İssa et al., only wedge resection was applied in 55 patients; segmental phenolisation in 53 patients; and phenol combined with wedge excision in 62 patients. The recurrence rates were 13% and 7.7% for patients treated with wedge resection and segmental phenolisation, respectively. Recurrence did not occur in patients treated with the combined method [16]. However, the addition of phenol to treatment is associated with successful outcomes and, rarely, serious complications due to its corrosive effects [20]. Several studies have shown that the application of electrocautery and cryotherapy after partial matrixectomy is associated with a lower rate of recurrence [21-24].

In several combined methods, several different complications (such as electrocautery-related infection and exudation, thermal damage due to CO_2_ laser, phenol-induced necrosis and periostitis, and digital nerve damage due to cryotherapy) can occur [25]. Although different methods are used in the treatment of ingrown toenails, certain recurrence rates are observed. As in similar studies, the suturing technique used in our research was effective in reducing recurrence, and the incidence of conditions such as post-operative necrosis, infection, and wound healing delay were low [26,27].

In our study, recurrence was not observed in the group that underwent surgery with mattress suturing. Meanwhile, the other group had a recurrence rate of 2%. However, the results did not significantly differ. In both groups, necrosis and corrosive injury did not occur due to the use of no chemicals. In addition, conditions such as the development of granulation tissue at the wound site, bleeding, and poor wound healing can develop. Moreover, it was observed that gender had no effect on recovery times and VAS score. In this study, 80% of patients in group 1 developed granulation tissue, and 20% in group 2 had mild granulation tissue. Therefore, although there was no difference in VAS values between the groups, we thought that granulation tissue had an effect on return to work.

## Conclusions

In ingrown toenail surgery, the cure rate is high if an appropriate approach is used. The advantage of the suturing technique in this study is the low recurrence rate as well as the absence of complications in combined methods. The incidence of conditions such as bleeding and granulation tissue was low. In addition, cosmetic satisfaction was high, and patients immediately returned to work/activities of daily living with this technique.
